# Bicycle Injury Prevention Education Using 360° Virtual Reality Experiences of Accidents and Computer-Based Activity

**DOI:** 10.3390/children9111623

**Published:** 2022-10-26

**Authors:** Woosup Lee, Ju Ok Park, Sung Ok Hong, Youngtaek Kim

**Affiliations:** 1 Department of Emergency Medicine, Hallym University College of Medicine Dongtan Sacred Heart Hospital, Hwaseong-si 18450, Korea; 2 Korean Health Information Management Association, Institute of Health and Medical Information, Seoul 05624, Korea; 3 Public Health Medical Service Office, Chungnam National University Hospital, Daejeon 34134, Korea

**Keywords:** child, adolescent, bicycle, health education, injury prevention, virtual reality

## Abstract

Injury prevention programs for children and adolescents need to be immersive and interactive. This study assessed a bicycle injury prevention program using technology-based education based on the Activated Health Education model and evaluated its effect on environmental factor awareness and attitude toward helmet-wearing. Using virtual reality technology, elementary and middle school students could experience simulated bicycle accidents. It was followed by an awareness phase that included a 30-min lecture where students self-learned and discussed risk-preventive factors. Students then developed user-created content and customized helmets they were given. We assessed students before the program, immediately afterward, and one month after the program. The number of respondents who said they were aware of surrounding bicycle lanes increased from 75.3% (pre-program) to 92.5% (one month after). Those who said they wore helmets often or always rose from 14.3% (pre-program) to 32.5% (one month later). The number needed to treat helmet-wearing behavior was approximately four, meaning that four people were required to participate in the program to have an impact on one person’s helmet-wearing. We found that virtual reality and computer-based activities can help children and adolescents experience bicycle accidents, be aware of risk factors, and change their behaviors responsibly.

## 1. Introduction

Cycling is currently being spotlighted as an economical, eco-friendly, short-distance sport for both transportation and leisure that also promotes health. However, cyclists are vulnerable when using roads, especially children and young people [1]. According to the International Road Traffic and Accident Database, the median share of cycling fatalities was 8% in 2016, up from 4% in 2000 [1,2]. Moreover, the annual reduction in death among cyclists was only 6.4%, while various other types of road deaths were reduced by 19.2% in 2020 because cycling became highly popular during COVID-19 lockdowns [3].

In addition, bicycle crashes are a common cause of traumatic brain injury (TBI) in children [4,5,6]. Helmets prevent head and face injuries, including brain injuries, in pediatric cyclists [7,8,9,10,11,12,13], but while helmet use is relatively high until the age of 10, it drops off afterward; helmets are used far less frequently by teenagers [5,14,15]. Reducing bicycle injuries in children requires a multifaceted approach to changing child behavior, increasing helmet adherence, and educating children on how to avoid hazardous situations [16,17].

It is essential to not only regulate the wearing of helmets by law but also instill the habit of wearing them from early childhood or adolescence [14,18]. There is a dramatic association between reports of increased helmet use with bicycle helmet legislation plus education rather than with legislation only [12,19,20,21]. Many community-based helmet programs for children and youth include the provision of education as well as free helmets [22]. There is evidence of a significant but minor effect in interventions that were purely rooted in education and did not provide free, subsidized, or discounted helmets [12,22]. Educational approaches often focus on helmet use; most bicycle injury prevention programs are school-based and use books, videos, and lecture formats to teach children general safety rules and information [16,23]. Such programs may also extend to bicycle skills training courses of varied duration, from one-day bicycle rodeos to long-term courses integrated into school curricula [23,24]. While educational and skills training bicycling programs may increase knowledge of cycling safety, they do not seem to translate into either a decreased injury rate or improved bicycle handling ability and attitudes. This could be due to children’s inability to transfer learned skills into real-life settings, an issue that has not been adequately studied [24]. 

Health education must be rooted in health behavior theories to develop, manage, and evaluate successful health education interventions. Of the many planning theories and models currently being used by health educators [25], the Activated Health Education (AHE) model was developed and refined based on a review of successful health education interventions to organize health content and improve health behavior [26]. In this model, there are three principles for enhancing health behavior among program participants: (1) the experiential phase: active involvement; (2) the awareness phase: awareness of positive and negative influences; and (3) the responsibility phase: facilitation of the identification and clarification of personal health values, and the development of a customized plan for behavioral changes [25,26].

In the experiential phase, individuals usually become aware of their actual health behavior through field studies, laboratory testing/screening, and surveys of targeted conduct. Previously, bicycle rodeos or skills training was used to gain experience, but they present problems with place, logistics, and safety. Virtual reality (VR) can be a potential alternative that provides a realistic bicycle accident experience without those problems [27]. VR offers several advantages over safety training: it provides a safe, computer-generated environment with realistic images and sounds that offer a feeling of immersion without risk of actual injury [28,29,30]; it provides opportunities for consistent feedback, practice, and repetition; it offers a motivating medium for children, and it provides wide accessibility through internet distribution [28]. Using these advantages, studies have been conducted on education with VR for child pedestrians [28,29,30,31,32,33].

Technology-based interventions, such as internet programs or software for injury prevention that incorporate VR, are not new. Most programs were designed for pediatric injury prevention, including pedestrian safety, fire safety, and dog-bite prevention [34]; however, no study has yet applied VR or technology-based intervention for bicycle injury prevention to this extent.

For this study, we aimed to evaluate the effect of a bicycle safety education program for children and adolescents based on the AHE model, using VR and computer-based activity, with measurements conducted immediately afterward and one month later. In addition, we hypothesized that children and adolescents who underwent this education program would show a significant improvement in their perception of bicycle helmet use behavior and environmental factors after undergoing this education program compared to before receiving it.

## 2. Materials and Methods

### 2.1. Study Design

In this study, we used the before and after study design to evaluate the effects of bicycle safety education programs.

### 2.2. Participants

For this study, we assessed an educational program that was developed in advance and conducted at a community center in a suburban part of South Korea. The community center organized all matters of the recruitment and operation of the participants following legal regulations, including the provision of informed consent for community education and the protection of personal information. The target audience was limited to children and adolescents from the fourth grade of elementary school to the third grade of middle school (aged 11 to 16 years old). Data were provided to the research team to assess the program’s effect after removing identifiable information and anonymizing it.

### 2.3. Procedures

**Figure 1:**
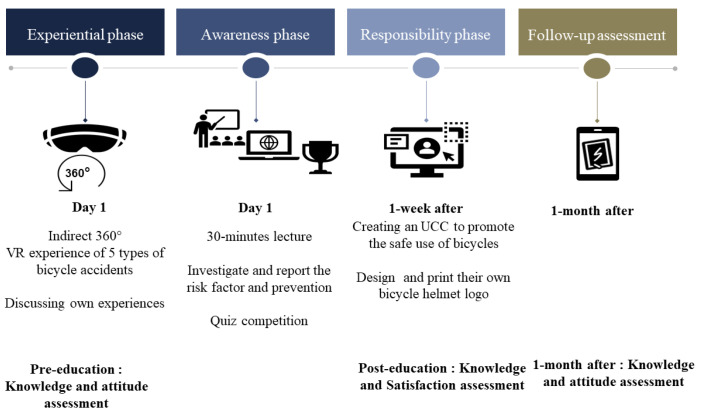
The bicycle injury prevention program based on the Activated Health Education (AHE) theory implemented by the community center.

**Figure 2:**
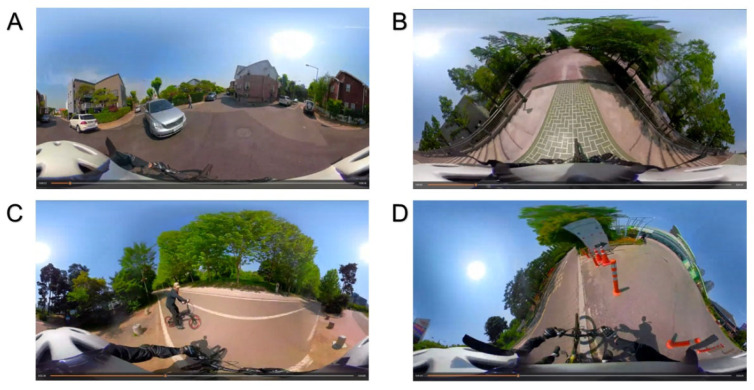
Example captures of virtual reality videos. (**A**) other vehicle accident, (**B**) accident in a downhill road, (**C**) other bicycle accident, (**D**) accident by the curbside.

The program was structured according to the AHE theory (Figure 1). For the experiential phase, five situations were presented through VR; accidents caused by pedestrians, other bicycles, vehicles (which are all risk factors while riding on roads), and accidents caused at curbsides and downhill roads, which are physical environmental risk factors. The videos were filmed by professional actors using a 360° camera (GoPro MAX, GoPro, Inc., San Mateo, California, USA) (Figure 2) and evaluated by adults for simulation appropriateness, reality, and motion sickness (Appendix A Table A1). The students used their smartphones and Google Cardboard to experience the VR safely on chairs. After watching the videos, each participant shared their impressions and their experiences.

The awareness phase began with a 30-min lecture. The purpose of the lecture was not to impart information such as traffic signs or cycling skills but to encourage students to assess related information to facilitate their engagement. Students then searched the internet to identify risk factors and discussed how to distinguish between environmental and host factors. Furthermore, they searched for and talked about ways to prevent bicycle accidents and presented their results and conclusions. In the end, the students took a quiz using Kahoot! (https://kahoot.com/, accessed on 13 July 2019).

In this program, the responsibility phase was implemented one week after the experiential and awareness phases, which were held in one day. The students developed user-created content (UCC) lasting from 1 to 3 min. The UCC was made and edited using Powtoon (https://www.powtoon.com/, accessed on 13 July 2019). The students also designed and printed logos expressing their personalities using LogoMakr (https://logomakr.com/, accessed on 13 July 2019), then customized the helmets they were given.

### 2.4. Data Collection

We conducted the pre-program assessment on the first day of the program. The post-program assessment was conducted at the end of the program (one week later). Another assessment, one month later, was sent to the respondents via mobile phone (Table 1, Figure 1).

Eighty students were recruited initially. Unexpectedly, it rained heavily on the first day of the program, so three students could not attend. Some students attended only the first day of the program, so they could not finish the post-program assessment, and we treated them as censored cases. After one month, the community center sent another assessment survey to the students who participated, with three reminder texts over one week. They were treated as censored cases if they did not respond within that period. For paired analysis, all censored cases were excluded because individual participants should have been observed longitudinally.

### 2.5. Data Analysis

All responses were expressed as the number and proportion of respondents. Knowledge tests were carried out before, immediately after, and one month after the program ended (Table 1). Questions about attitudes were asked before the program and one month afterward (Table 1). Attitude-related questions were compared using the McNemar test. The outcomes included the odds ratios (ORs) and 95% confidence intervals (CIs). In addition, we estimated the risk difference (RD) before and after the program, and we calculated the number needed to treat (NNT). We used Stata version 16 for data analysis. Additionally, we described the content of UCC through the narrative description.

## 3. Results

Of the group, 77, 68, and 40 students responded to the assessments before the program, immediately afterward, and one month later, respectively. Of these, there were 37 (48.1%), 35 (51.5%), and 27 (67.5%) elementary school students, respectively. In the pre-program assessment, 63 students (81.8%) said they owned a bicycle, and 41 (53.3%) said they had a helmet. In other words, only 65% of student bicycle owners had a helmet. When comparing across grades, 67.6% of elementary and 40.0% of middle school students said they had helmets; the higher the grade, the lower the rate of helmet ownership. When asked about experiences of bicycle-related injuries in the past month, 14.3% of the students had experienced injuries in the pre-program assessment, while only 5% experienced injuries in the assessment one month later.

To determine how aware students were of environmental factors for bicycling, we asked them whether they knew of bicycle lanes in the neighborhood. The number of respondents aware of bicycle lanes increased from 75.3% pre-program to 92.5% one month later. When asked about the frequency of wearing helmets, the proportion of respondents who said they wore them often or always rose from 14.3% pre-program to 32.5% one month later (Table 2).

The percentage of correct answers to the questions about types of bicycle lanes was maintained at 88.3% (pre-program), 89.7% (post-program), and 90.0% (one month later). The percentage of correct answers to the question on how to use the crosswalk with a bicycle remained high at 97.4% (pre-program), 95.6% (post-program), and 100% (one month later). The percentage of correct answers to the questions about the bicycle pre-inspection rose from 68.8% (pre-program) to 86.8% (post-program), and 95.0% (one month later). The percentage of correct answers to the question about traffic signs related to bicycles rose from 11.7% (pre-program) to 39.7% (post-program), and 30.0% (one month later), which was lower than the percentage of correct answers to other questions.

The students appeared to be satisfied with the program. By grade, overall satisfaction among middle school students was lower than that of elementary school students. When asked about the degree to which they learned new things about bicycle safety, the average score of the respondents was 7.7 out of 10 (Table 2).

As a result of a paired analysis of the 40 students who responded to both the assessment before the program and the assessment one month afterward, the number of respondents who frequently or always wore a helmet when using a bicycle rose significantly (*p*-value = 0.0225) about 5.5 times (OR: 5.5, 95% CI: 1.20–51.07), and the risk difference was 0.26 (95% CI: 0.046–0.469). The NNT for helmet-wearing was about 4, indicating that four students needed to be educated to cause one more student to wear a helmet (Table 3).

The research team and instructors reviewed the content of the UCC to establish the degree to which students had developed a sense of responsibility. Most of the students included risk factors for bicycle accidents in their UCC. Factors related to cyclists (host factors) that were commented on included not wearing protective equipment (helmets), riding in the wrong direction on the road, jaywalking on a bicycle, riding in places other than bicycle lanes, and lack of knowledge of traffic signs or violations of traffic rules. Students also mentioned appropriate clothing, proper helmet-wearing, and pre-inspection methods (tires, brakes, chains, headlights, steering wheels, etc.). Weather (rain or snow), road surface conditions, and illegal bike lanes were mentioned as environmental factors. Some students referred to legal regulations related to bicycle lane use, proper bicycle parking, and dealing with an accident.

## 4. Discussion

We aimed to investigate whether a program rooted in VR and computer-based education based on the AHE model could improve bicycle safety for elementary and middle school students. According to the assessment outcomes, the participants’ knowledge of bicycle safety and their awareness of the surrounding bicycle environment were improved or maintained. In addition, the rate of wearing a bicycle helmet increased. Students evenly recognized and presented host and environmental factors related to bicycle accidents in the context of UCC. Students reinforced their sense of responsibility through this program to investigate and self-learn safety behaviors for accident prevention.

Our results support that technology-based injury prevention programs influence behavior change. Recently, programs using digital technology have been introduced in health education [34,35]. Experiential and participatory education using digital technology, rather than one-sided lecture-style education, consistently improve children’s knowledge and behavioral outcomes [34].

In this program, students can experience VR using a smartphone and Google Cardboard. This experience suggests that logistics problems can be solved more easily than other bicycle riding experiences. Indeed, there was heavy rain on the first day of the program, but the participants experienced riding a bicycle on a sunny day through VR. The Google Cardboard format we used has the potential for large-scale distribution asmost smartphones have the technology to be incorporated into a simple, cheap headset to display content in the VR format. Content is distributed easily via YouTube or mobile applications. The availability of consumer-level 360° cameras is also making it easier for amateur filmmakers to create and distribute 360° footage for viewing within a VR headset [36].

The indirect experience of using VR has the advantage of fewer time or place limitations compared to direct bicycle riding. VR education can expose the user to risk factors without being vulnerable to injury, thereby boosting the learners’ cognition and presenting various environments through digital manipulation [33]. VR offers the same unlimited opportunities without placing children at risk in real traffic, such as an intervention conducted by teachers or parents in a one-on-one model in the actual road environment [32]. Previous studies have shown VR to be a promising tool for teaching pedestrian safety [27,28,29,32,37]. We found a similar effect of VR on bicycle injury prevention in children and adolescents.

The program’s goal was to raise students’ awareness of behavioral changes. When comparing the pre-program and one-month later assessments, it was found that helmet-wearing had significantly improved. There was a positive relationship with helmet ownership, and previous research recommended helmet promotion development, including social marketing strategies to maximize consumer acceptance for middle school students [38].

A meta-analysis published in 2011 [22] revealed that, non-legislative intervention seems to be effective in self-reported helmet wearing in those receiving the intervention (OR 3.27, 95% 1.56 to 6.87) compared to those receiving no intervention. Moreover, compared to studies providing education only (OR 1.93, 95% CI 1.03 to 3.63), those providing free helmets were much more effective (OR 7.27, 95% CI 1.28 to 41.44). In this study, we applied the educational element with free helmet provision. Then the number of respondents who frequently or always wore a helmet when using a bicycle rose significantly by about 5.5 times, which is consistent with the previous study.

There was a significantly greater knowledge of helmet and safety rules and the ability of hazard discrimination for the intervention group to the control group in the school-based experiment using the eHealth product, which utilizes video, animation, and images to train children [16]. In another previous study of the school and summer camps program, nearly 55% of students improved, and almost 15% retained their knowledge test scores in both groups [23]. We obtained similar results in this study. The knowledge of safetyimproved and was maintainedfor one month. However, most of the students who participated in this study already knew well how ride a bicycle on traffic roads (Q7), the bicycle checklist for safe driving (Q8), and the best way to use a pedestrian crosswalk by bike (Q9) before the program. For the traffic sign related to bicycle riding, only 11.7% of students knew the correct meaning in the pretest and 39.7% in the post-test. There was no lecture about traffic signs, but students searched the information on traffic signs if they needed to make UCC. It reflected that the children and students less significantly considered the knowledge of traffic signs as a factor in bicycle safety.

In the present study, when the children made and printed their helmet logos, they reinforced their sense of responsibility for their safety. Moreover, students had to confirm the safe behavior or skill of bicycling and attain a level of cognition that could explain the host factors of bicycle injury (both to themselves and others) to provide accurate information in their UCC. These activities could encourage helmet use for reasons other than safety and link helmet ownership to behaviors. 

Another goal was to raise awareness of environmental factors. In the UCC developed by the students, we found an increase in students’ awareness of the risk factors in the cycling environment. According to the assessment, one month after the program ended, the students’ awareness of the location of the surrounding bicycle lanes increased. However, it was difficult to determine the accuracy of the responses because it was not possible to conduct a field survey.

Since the goal of this program was not to deliver knowledge or information, the understanding of traffic laws and road signs did not increase. The students were encouraged to search for data and obtain information independently, but they found it difficult to acquire knowledge about road signs and traffic rules. Regarding the questions about bicycle signs and hand signals, the percentage of correct answers increased according to the outcomes of all assessments; however, the percentage was very low overall. This is because students have little experience learning about bicycle-related laws or signs. Moreover, information on traffic signs and rules is not commonly found in the online content that teenagers encounter. In a previous study on the effect of pedestrian safety education for children using VR, children trained within a virtual environment showed improvement in pedestrian behavior but not in knowledge about pedestrian safety [39]. In another systematic review, computer-based communication using kiosks effectively delivered information, and VR programs improved behaviors, but there were few gains in knowledge [34]. Our findings are also consistent with previous studies.

While the overall results of the program evaluation are highly promising, there are several limitations. First, this program was designed not for research but for implementation at a community center. For this reason, there was no control group used to evaluate the program’s effectiveness. However, the pre-and post-assessment revealed the efficacy of the new approach to injury prevention education for children and adolescents. Second, the students participated in the program voluntarily, which could have led to a biased population already interested in computer-based education or safety issues. The small number of participants is also a limitation that makes it difficult to generalize the findings. In previous studies using VR, the number of participants varied widely, from a study of 44 [31] to as many as 240 [39]. Due to the community center’s facility, one training session was limited to 20 people or less. In addition, given the learning ability of elementary school students, each program was conducted for less than three hours a day, for a total of two days, with a one-week interval. The requirement of participating in two all-day programs made it difficult to recruit students. In addition, this scheduling constraint made it challenging to recruit instructors. If a resident teacher at a school or community center runs this program regularly, it will be possible to educate numerous students effectively.

## 5. Conclusions

There is significant potential for technology-based education programs, through computer or mobile technology, to be used for injury prevention. Based on robust health behavior or education theory, technology can be powerful in affecting behavior change and awareness of environmental factors. The evaluation of technology-based education (VR, internet, and UCC) based on the AHE model for bicycle injury prevention in children and adolescents indicated that participants’ attitudes toward wearing bicycle helmets and their perception of a safe environment were significantly improved. As technology improves and evidence grows, such interventions should be implemented and disseminated widely.

## Figures and Tables

**Table 1:** Questions on the assessments: Before the program, immediately after the program, and one month later.

Categories	Questions	Choices or Score	Pre-Program	Post-Program	One Month Later
General	Q1. Do you have a bicycle?	Yes/No	A	NA	NA
	Q2. Do you have a bicycle helmet?	Yes/No	A	NA	NA
	Q3. Have you ever suffered more than a bruise or abrasion in a bicycle accident in the past month?	Yes/No	A	NA	A
Attitude	Q4. Do you know where there is a bicycle lane near your house?	Yes, I know/No, I do not know	A	NA	A
	Q5. How often have you used your helmet in the past month?	Never or not often/Often or always	A	NA	A
Knowledge	Q6. What does this traffic sign mean? (MCQ with picture)		A	A	A
	Q7. Which lane of the multi-lane driving roadcan be used for cycling in this picture? (MCQ with picture)		A	A	A
	Q8. Which of the following is not on a bicycle checklist for safe driving? (MCQ)	(1) To make sure both brakes are working	A	A	A
		(2) To ensure maximum visibility by raising the saddle as high as possible. (Correct answer)			
		(3) To check that the bicycle tire pressure is adequate			
		(4) To lubricate the chain to operate smoothly and prevent rust			
	Q9. Which of the following is the best way to use a pedestrian crosswalk by bike? (MCQ)	(1) To get off and drag your bike. (Correct answer)	A	A	A
		(2) To slow down but just ride the bike			
		(3) While riding a bicycle, cross the road next to the pedestrian crossing.			
		(4) Bicycles cannot be used in the crosswalk.			
Satisfaction with the program	Q10. Was this program fun?	1 point (dissatisfied) - 10 points (satisfied)	NA	A	NA
	Q11. Did you learn anything new about bicycle safety through this training?	1 point (dissatisfied) - 10 points (satisfied)	NA	A	NA
	Q12. Are you willing to recommend this program to your friends?	1 point (dissatisfied) - 10 points (satisfied)	NA	A	NA

Note: A, Applicable; NA, Not applicable; MCQ, Multiple choice question.

**Table 2:** Responses: Before the program, immediately after the program, and one month later.

Categories	Questions/Answers	Pre-Program			Post-Program			One Month Later		
		Elementary	Middle	Total	Elementary	Middle	Total	Elementary	Middle	Total
		*N* = 37	*N* = 40	*N* = 77	*N* = 35	*N* = 33	*N* = 68	*N* = 27	*N* = 13	*N* = 40
General ^a^	Q1/Yes	31 (83.8)	32 (80.0)	63 (81.8)	-	-	-	-	-	-
	Q2/Yes	25 (67.6)	16 (40.0)	41 (53.3)	-	-	-	-	-	-
	Q3/Yes	8 (21.6)	3 (7.5)	11 (14.3)	-	-	-	1 (3.7)	1 (7.7)	2 (5.0)
Attitude ^a^	Q4/Yes, I know	30 (81.1)	28 (70.0)	58 (75.3)	-	-	-	25 (92.6)	12 (92.3)	37 (92.5)
	Q5/Often or always	8 (21.6)	3 (7.5)	11 (14.3)	-	-	-	9 (33.3)	4 (30.8)	13 (32.5)
Knowledge ^a^	Q6/Correct answer	3 (8.1)	6 (15.0)	9 (11.7)	11 (31.4)	16 (48.5)	27 (39.7)	8 (29.6)	4 (30.8)	12 (30.0)
	Q7/Correct answer	32 (86.5)	36 (90.0)	68 (88.3)	32 (91.4)	29 (87.9)	61 (89.7)	25 (92.6)	11 (84.6)	36 (90.0)
	Q8/Correct answer	26 (70.3)	27 (67.5)	53 (68.8)	28 (80.0)	31 (93.9)	59 (86.8)	26 (96.3)	12 (92.3)	38 (95.0)
	Q9/Correct answer	36 (97.3)	39 (97.5)	75 (97.4)	33 (94.3)	32 (97.0)	65 (95.6)	27 (100.0)	13 (100.0)	40 (100.0)
Evaluating this program ^b^	Q10	-	-	-	9.2 ± 1.43	7.8 ± 1.58	8.5 ± 1.65	-	-	-
	Q11	-	-	-	8.0 ± 1.90	7.3 ± 1.85	7.7 ± 1.89	-	-	-
	Q12	-	-	-	8.5 ± 1.90	7.8 ± 1.94	8.1 ± 1.94	-	-	-
Total		37 (100)	40 (100)	77 (100)	35 (100)	33 (100)	68 (100)	27 (100)	13 (100)	40 (100)

Note: ^a^ The questions were Yes/No or multiple-choice. All results are presented with the number and proportion of respondents. ^b^ Questions were answered using a 10-point Likert scale. All results are presented with the means and standard deviations.

**Table 3:** Changes in attitudes toward wearing a helmet and environmental awareness.

			Pre-program			
			Often or always	Not often	Total	*p*-value
Helmet wearing	One month later	Often or always	2	11	13	0.0225
		Not often	2	20	22	
		Total	4	31	35	
	Odds ratio (1 month later vs. pre-program)			5.5 (95% CI: 1.20, 51.07)		
	Risk difference			0.26 (95% CI: 0.046, 0.469)		
	NNT			4		
			Pre-program			
			Yes, I know.	No, I do not know.	Total	*p*-value
Recognition of bicycle lanes near house	One month later	Yes, I know.	27	8	35	0.0391
		No, I do not know.	1	1	2	
		Total	28	9	37	
	Odds ratio (one month later vs. pre-program)			8.0 (95% CI: 1.07, 354.98)		
	Risk difference			0.19 (95% CI: 0.015, 0.363)		
	NNT			5		

Note: NNT, number needed to treat; CI, confidence interval.

## Data Availability

Restrictions apply to the availability of these data. Data was obtained from Narewul Social Welfare Center and are available from the authors with the permission of the Narewul Social Welfare Center.

## Appendix A

**Table A1:** Evaluation of a 360° virtual reality videos.

Categories	Items	VR1: Pedestrian Accident	VR2: Other Bicycle Accident	VR3: Other Vehicles Accident	VR4: Curbside	VR5: Downhill Road
Simulator appropriateness	Scenarios in this video are a common occurrence. ^a^	4.4 (0.72)	3.7 (1.10)	3.9 (1.01)	3.6 (1.33)	4.0 (1.07)
	I actually experienced an accident like this video. ^b^	6 (24.0)	4 (16.0)	23 (92.0)	5 (20.0)	8 (32.0)
Simulator reality	The surrounding environment of this video feels realistic. ^a^	3.7 (1.08)	4.0 (0.93)	3.9 (0.97)	3.7 (1.20)	3.7 (0.99)
	The sound of this video feels real. ^a^	3.4 (1.10)	3.5 (1.22)	3.8 (1.00)	3.8 (1.19)	3.8 (1.11)
	The screen of this video feels like it is actually falling. ^a^	3.4 (1.14)	3.7 (1.11)	3.3 (1.18)	3.6 (1.14)	3.9 (1.08)
Simulator sickness	I felt nauseous while watching or after watching this video. ^a^	2.9 (1.57)	2.8 (1.57)	2.1 (1.06)	2.3 (1.24)	2.4 (1.22)
	I felt dizzy while watching or after watching this video. ^a^	2.5 (1.35)	2.3 (1.19)	2.1 (1.10)	2.0 (1.05)	2.1 (1.13)
	I felt a visual disturbance while watching or after watching this video. ^a^	1.8 (1.07)	2.1 (1.39)	1.8 (1.19)	1.9 (1.25)	2.0 (1.36)

Notes: ^a^ Questions were answered using a 10-point Likert scale. All results are presented with the means and standard deviations. ^b^ This was a Yes/No choices question. All results are presented with the number and proportion of respondents.
